# SARS-CoV-2 spike protein promotes IL-6 trans-signaling by activation of angiotensin II receptor signaling in epithelial cells

**DOI:** 10.1371/journal.ppat.1009128

**Published:** 2020-12-07

**Authors:** Tapas Patra, Keith Meyer, Lizzie Geerling, T. Scott Isbell, Daniel F. Hoft, James Brien, Amelia K. Pinto, Ratna B. Ray, Ranjit Ray

**Affiliations:** 1 Department of Internal Medicine, Saint Louis University, Missouri, United States of America; 2 Department of Molecular Microbiology & Immunology, Saint Louis University, Missouri, United States of America; 3 Department of Pathology, Saint Louis University, Missouri, United States of America; Icahn School of Medicine at Mount Sinai, UNITED STATES

## Abstract

Cytokine storm is suggested as one of the major pathological characteristics of SARS-CoV-2 infection, although the mechanism for initiation of a hyper-inflammatory response, and multi-organ damage from viral infection is poorly understood. In this virus-cell interaction study, we observed that SARS-CoV-2 infection or viral spike protein expression alone inhibited angiotensin converting enzyme-2 (ACE2) receptor protein expression. The spike protein promoted an angiotensin II type 1 receptor (AT1) mediated signaling cascade, induced the transcriptional regulatory molecules NF-κB and AP-1/c-Fos via MAPK activation, and increased IL-6 release. SARS-CoV-2 infected patient sera contained elevated levels of IL-6 and soluble IL-6R. Up-regulated AT1 receptor signaling also influenced the release of extracellular soluble IL-6R by the induction of the ADAM-17 protease. Use of the AT1 receptor antagonist, Candesartan cilexetil, resulted in down-regulation of IL-6/soluble IL-6R release in spike expressing cells. Phosphorylation of STAT3 at the Tyr705 residue plays an important role as a transcriptional inducer for SOCS3 and MCP-1 expression. Further study indicated that inhibition of STAT3 Tyr705 phosphorylation in SARS-CoV-2 infected and viral spike protein expressing epithelial cells did not induce SOCS3 and MCP-1 expression. Introduction of culture supernatant from SARS-CoV-2 spike expressing cells on a model human liver endothelial Cell line (TMNK-1), where transmembrane IL-6R is poorly expressed, resulted in the induction of STAT3 Tyr705 phosphorylation as well as MCP-1 expression. In conclusion, our results indicated that the presence of SARS-CoV-2 spike protein in epithelial cells promotes IL-6 trans-signaling by activation of the AT1 axis to initiate coordination of a hyper-inflammatory response.

## Author summary

The mechanisms of SARS-CoV-2 induced excessive inflammatory response associated with disease severity needs to be critically studied for therapeutic intervention. Cytokine storm is typical of macrophage activation; which leads to tissue damage, lung injury, and acute respiratory distress syndrome. Lung epithelial cells; a primary host for SARS coronavirus infection, can generate a cytokine response leading to an expanded pathology. Our present study was designed to understand SARS-CoV-2 and human epithelial cell interactions, by delineating their contribution to inflammatory cytokine systems with special emphasis on ectopic expression of the viral spike protein. We observed that SARS-CoV-2 infection or spike protein expression in human epithelial cells inhibits ACE2 expression leading to the induction of AT1 signaling, and promotion of IL-6/soluble IL-6R release. IL-6 is one of the major mediators of the hyper-inflammatory response, and can exert signaling through two main pathways referred to as classical or trans- signaling to coordinate prominent pro-inflammatory properties. Our experimental observations suggest the potential for IL-6 trans-signaling during SARS-CoV-2 infection of epithelial cells. These observations will aid in the development of new therapeutic modalities for combating COVID-19 associated disease severity. Aggressive tissue damage via increased IL-6 secretion and other cytokines leading to a hyper-inflammatory response are in line with COVID-19 disease severity. We propose that SARS-CoV-2 infection in pulmonary epithelial cells induces IL-6 trans-signaling for secretion of chemokines; like MCP-1, from pulmonary vascular endothelial cells to attract monocytes/macrophages and create a hyper-inflammatory state leading to enhanced disease severity and acute respiratory distress syndrome.

## Introduction

The novel human coronavirus causative agent of coronavirus disease-19 (COVID-19) initiates and promotes rapid disease spread and severity. Potential mechanisms for severe acute respiratory syndrome coronavirus 2 (SARS-CoV-2) disease progression include a high rate of viral replication, resulting in enhanced host cell cytolysis, as well as, production of inflammatory cytokines and chemokines that perpetuate damage [1]. In addition, accumulation of monocytes, macrophages and neutrophils may also exacerbate disease progression. Our current understanding links COVID-19 with the development of a maladaptive immune response where multi-organ dysfunction may coincide with a severe disease state. The pathological mechanisms involved in multi-organ damage caused by SARS-CoV-2 infection are not well understood. However, widespread distribution of macrophages throughout organs or systemic virus infection may be contributing factors for underlying multi-organ dysfunction due to macrophage activation and infiltration occurring in the lungs of COVID-19 patients, who are known to secrete high levels of inflammatory cytokines [2].

Acute kidney injury, cardiac damage, and abdominal pain are the most commonly reported side effects of COVID-19 [3, 4]. SARS-CoV-2 may productively infect these organs, or damage could result from specific pathogenic conditions, including cytokine release syndrome [5]. Inflammasome activation or pyroptosis and production of cytokines/chemokines by infected cells may play important roles in virus-associated pathogenesis.

Recent clinical studies have found that COVID-19 patients with severe illness had elevated levels of certain cytokines including IL-6, TNF-α, IL-10, MCP-1 and IP-10 [6–8]. The pathophysiology of COVID-19 is far from being understood, and the absence of an effective medical regimen has led to the development of new therapeutic strategies based on pathophysiological assumptions. IL-6 is an important inducer of the acute phase SARS-CoV-2 infection hyper-inflammatory response [9, 10]. Tocilizumab (TCZ) is a well-established IL-6 receptor monoclonal antibody that inhibits both the classical and trans-signaling axis widely used for the treatment of rheumatoid arthritis. Several clinical outcomes suggested that an early use of tocilizumab is beneficial for survival, reduction of hospitalization stay, and duration of oxygen support in COVID-19 patients [11–13].

The angiotensin converting enzyme (ACE) and its close homologue ACE2 belong to the dipeptidyl carboxypeptidase enzymatic family. ACE converts angiotensin I into the peptide angiotensin II, which binds to and activates the AT1 receptor leading to vasoconstriction. On the other hand, ACE2 cleaves angiotensin II and produces angiotensin 1–7 peptide leading to vasodilation via activation of its G protein coupled Mas receptor [14]. ACE2 is highly expressed in human lung tissue, and is identified as the prime entry receptor for SARS-CoV-2 virus [15]. Relatively little is known about the effect of SARS-CoV-2 virus binding to ACE2 may modulate ACE2 enzymatic activity, impacting its utility for maintaining physiological homeostasis [16]. The STAT3 transcription factor is an important signaling molecule for many cytokines and growth receptors. Phosphorylation of the specific amino acid residue (Tyr705) in response to ligand stimulation determines STAT3 function. Phosphorylation at Tyr705 induces STAT3 dimerization, nuclear translocation, and promotes transcriptional activation for cytokine synthesis. However, phosphorylation of STAT3 at Ser727 through the MAPK pathways produces anti-apoptotic activities associated with its mitochondrial translocation [17, 18].

IL-6 is a pleiotropic cytokine with biological functions that affect tissues beyond the immune system and the vasculature [19]. Binding of IL-6 to the transmembrane receptor induces the homo-dimerization of a signal-transducing component, gp130, followed by tyrosine specific phosphorylation of STAT3, and its translocation to the nucleus for generation of inflammatory responses. Signaling via membrane-bound IL-6R is denoted as classic signaling. On the other side, a soluble form of IL-6 receptor truncated at the integral membrane portion can also bind IL-6 with a similar affinity as its transmembrane receptor. The complex of IL-6 and soluble IL-6R can bind to gp130 on cells, which do not express the IL-6R, and which are unresponsive to IL-6. This process has been termed as IL-6 trans-signaling [20, 21].

Our present study was designed to understand SARS-CoV-2 and host cell interactions, with special emphasis on the inflammatory cytokine system. Ectopic expression of viral spike protein was also analyzed in understanding potential mechanisms of SARS-CoV-2 interaction with cells. We observed that SARS-CoV-2 infection or spike protein expression in human epithelial cells inhibits ACE2 expression and promotes IL-6/soluble IL-6R release. We further defined the influence of IL-6 trans-signaling that may have upon neighboring endothelial cells.

## Results

### ACE2 expression is inhibited upon SARS-CoV-2 infection or spike gene transfection

**Fig 1:**
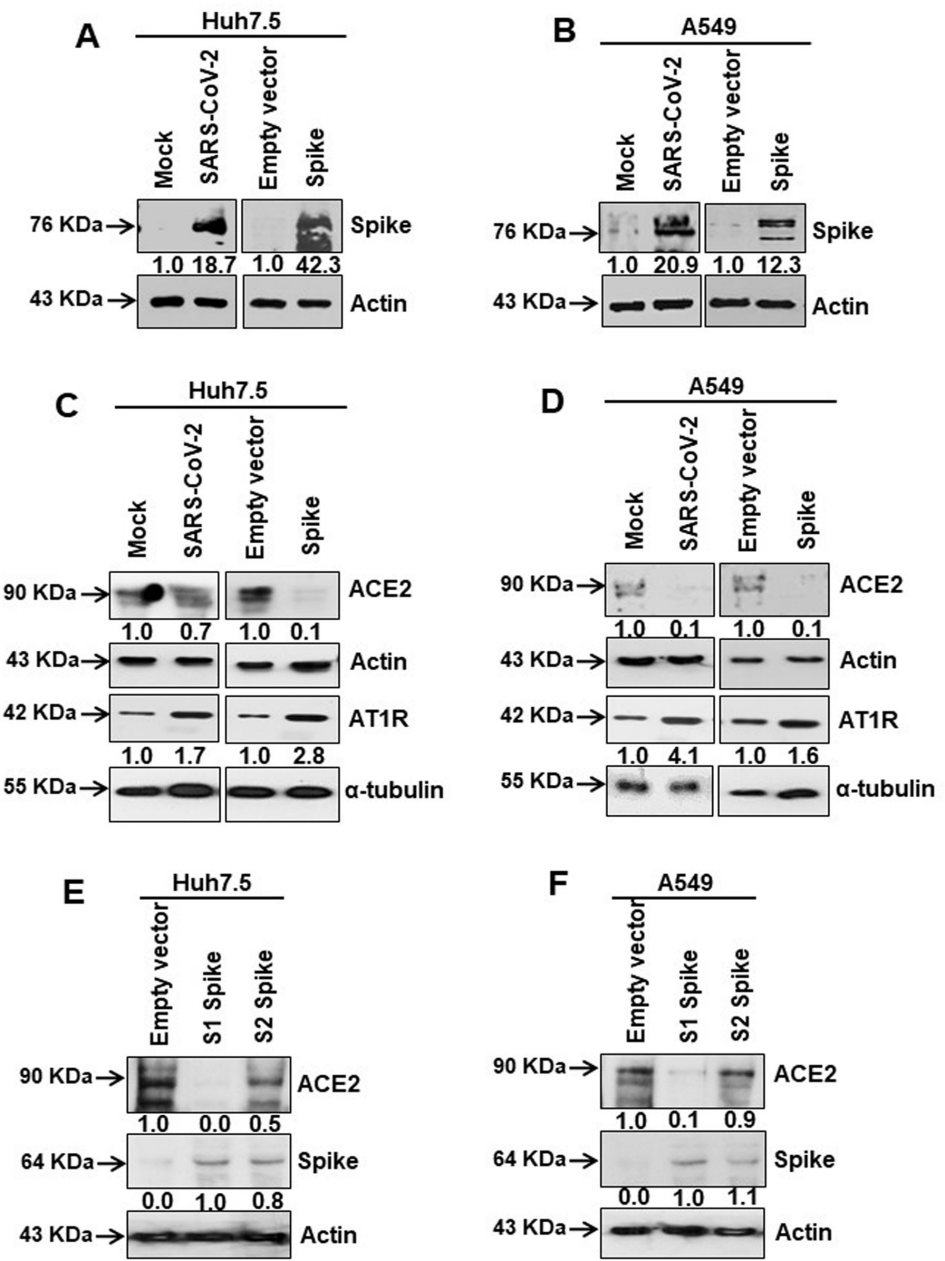
SARS-CoV-2 spike protein inhibits ACE2 expression. **(A, B)** Western blot analysis of SARS-CoV-2 spike protein expression in Huh7.5 and A549 cell lysates prepared after 48 h of mock- or SARS-CoV-2 virus infection, or transiently transfected with an empty vector or SARS-CoV-2 spike gene construct. **(C, D)** Western blot analysis of ACE2 and AT1 receptor expression in Huh7.5 and A549 cell lysates prepared after 48 h of mock- or SARS-CoV-2 virus infection, or transiently transfected with empty vector or SARS-CoV-2 spike gene construct. **(E, F)** Western blot analysis of ACE2 expression in Huh7.5 and A549 cell lysates prepared after 48 h of transfection of empty vector or SARS-CoV-2 spike S1 or S2 gene construct. Expression level of actin or tubulin in each lane from the same gel is shown as a total protein load for comparison.

We used Huh7.5 and A549 cells by infection with SARS-CoV-2 or transient transfection with cloned viral spike gene. Microscopically we noted minimal indications of cell death (<10%) 48 h post-infection or transfection. Spike protein expression status was analyzed from cell lysates generated after 48 h (Fig 1A and 1B). To identify specific receptor associated effects brought about by SARS-CoV-2 infection or the expression of spike protein, cell lysates were analyzed for ACE2 and AT1 receptor (AT1R) expression status by western blot. ACE2 protein expression was significantly reduced in virus infected or spike gene transfected lung epithelial cells, while AT1R expression was significantly increased in both infected and transfected cell lysates (Fig 1C and 1D). ACE2 suppression by S1 (1–685 aa) or S2 (686–1273 aa) regions of the spike protein was further analyzed (Fig 1E and 1F). Cells transfected with the S1 region of spike protein, but not the S2 region, displayed significantly reduced levels of ACE2 expression. Our results suggested that SARS-CoV-2 spike protein promotes inhibition of ACE2 expression, and induction of AT1 signaling.

### SARS-CoV-2 spike protein mediated activation of AT1 signaling induces MAPK/NF-κB axis

**Fig 2:**
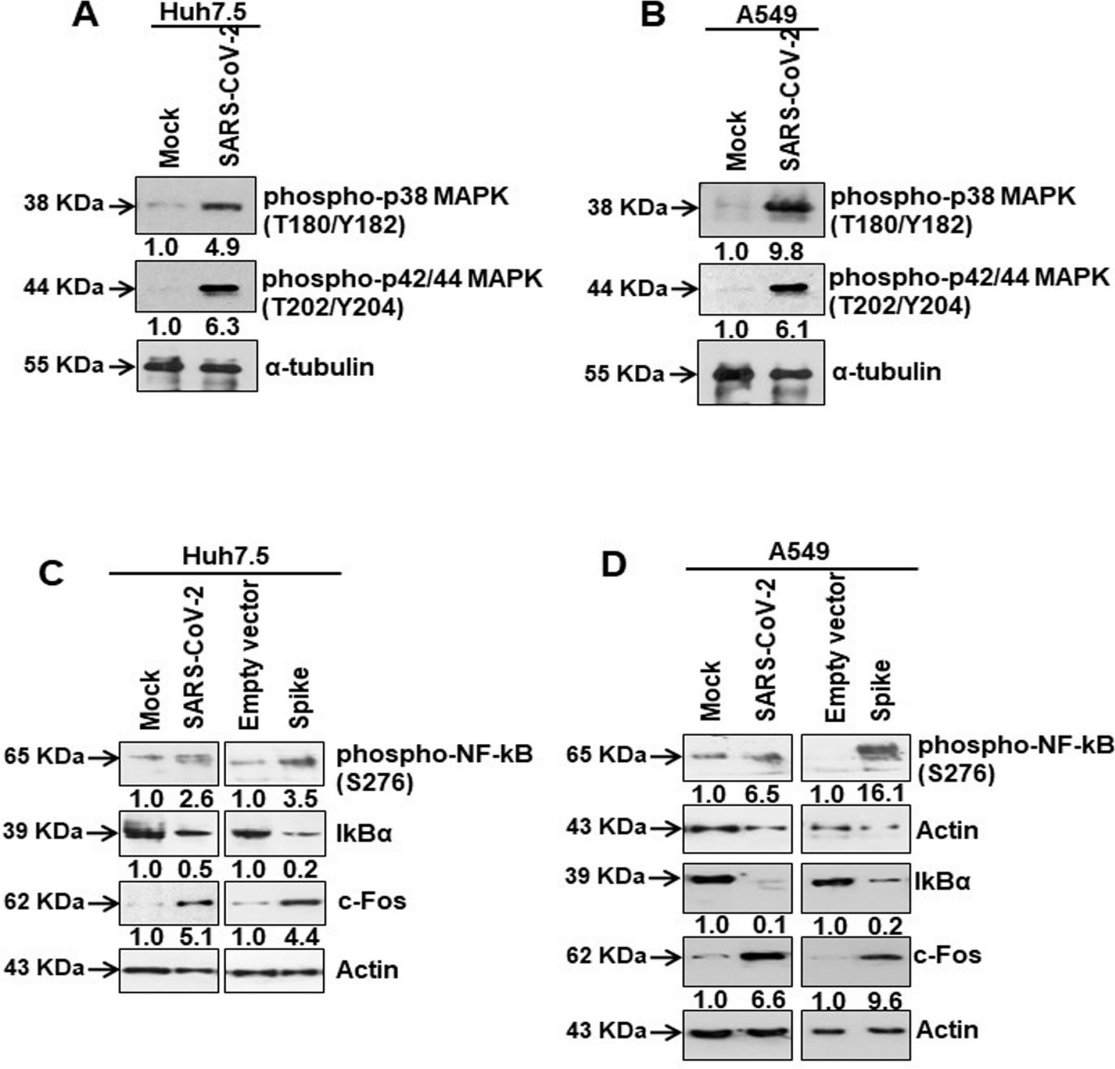
SARS-CoV-2 spike protein activates the transcription factors for IL-6 synthesis. **(A, B)** Western blot analysis of phospho-p38 MAPK (Thr180/Tyr182) and phospho-p42/44 MAPK (Thr202/Tyr204) in Huh7.5 and A649 cell lysates prepared after 48 h of mock- or SARS-CoV-2 virus infection, or transiently transfected with empty vector or SARS-CoV-2 spike gene construct. **(C, D)** Western blot analysis of phospho-NF-κB (Ser276), IκBα and c-Fos expression in Huh7.5 and A549 cell lysates prepared after 48 h of mock- or SARS-CoV-2 virus infection, or transiently transfected with empty vector or SARS-CoV-2 spike gene construct. Expression level of actin or tubulin in each lane from the same gel is shown as a total protein load for comparison.

We examined the possibility that AT1 upregulation may play a role in MAPK and NF-kB activation in virus infected or spike gene transfected cells generated from two distinct human tissues (Huh7.5 and A549). We observed that SARS-CoV-2 infected Huh7.5 and A549 cells expressed an elevated level of phospho-p38 MAPK (Thr180/Tyr182) and phospho-p42/44 MAPK (Thr202/Tyr204) molecules (Fig 2A and 2B). MAPK regulates p65/NF-κB activation for cytokine synthesis [22]. We observed enhanced phosphorylated NF-κB (Ser276), while the level of IκBα decreased (Fig 2C and 2D). Phosphorylation at Ser276 of NF-κB and degradation of IκBα indicates transcriptional activation of nuclear p65/NF-kB. c-Fos, a protein involved in AP-1 transcriptional regulation, is induced by a variety of cellular stresses that induce one or more of the MAPK pathways [23, 24]. Therefore, we analyzed viral spike transfected cells to identify if there is c-Fos induction following MAPK activation. Virus infection or spike protein expression enhanced c-Fos level in both Huh7.5 and A549 cell types (Fig 2C and 2D) which could act as a transcriptional regulator. To examine the activity of AP-1 transcriptional factor complex [25], we performed a functional reporter assay using AP-1 binding sites with minimal promoter and luciferase gene, and at varying doses of vector or SARS-CoV-2 spike constructs for 24 or 48 hours. We did not observe AP-1 transcription complex associated luciferase activation by spike protein under our experimental conditions. Further work is necessary to elucidate the role of AP-1 complex as transcriptional regulator.

**Fig 3:**
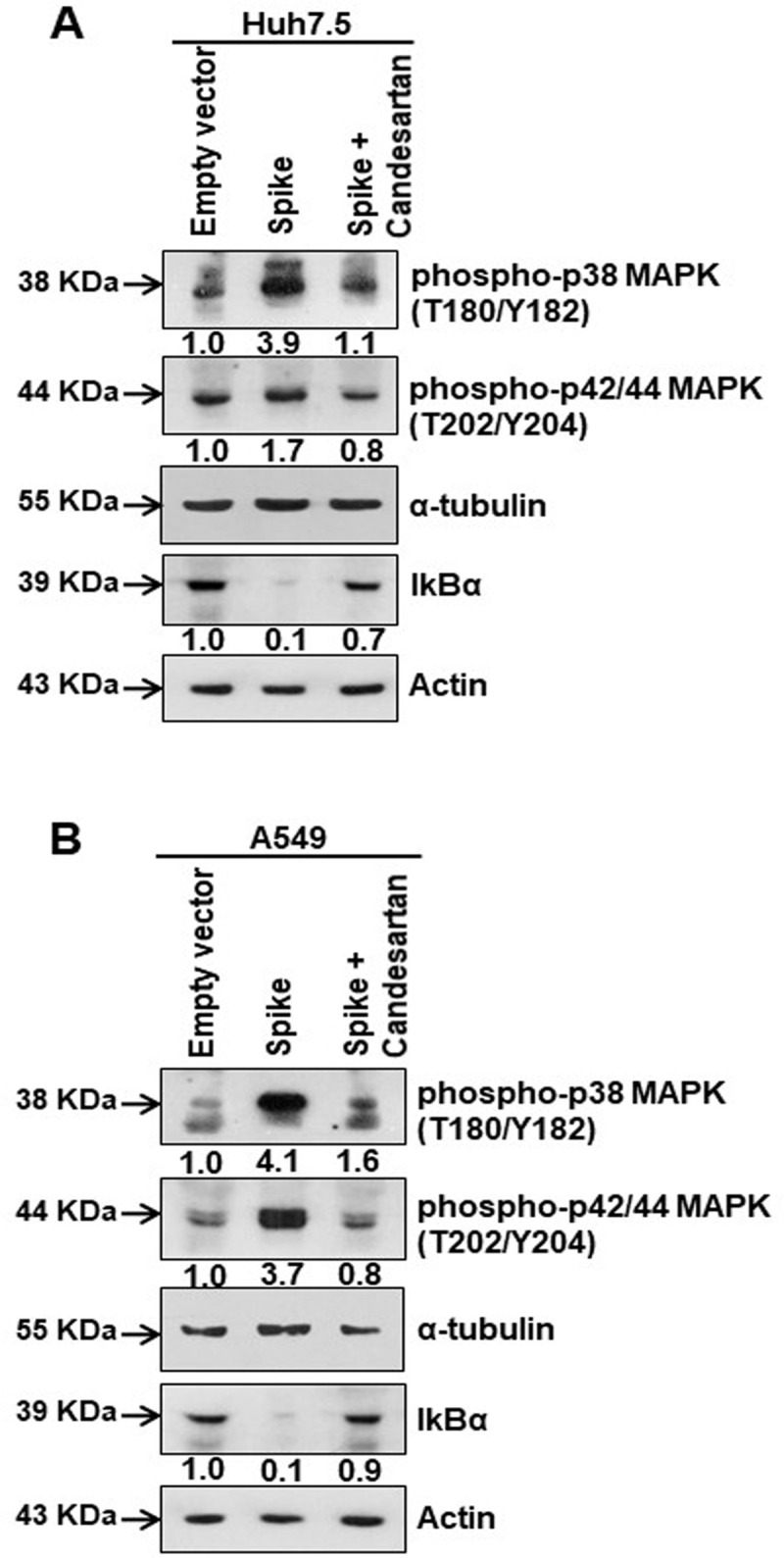
Candesartan cilexetil as an AT1 receptor antagonist prevent MAPK activation. **(A)** Western blot analysis of phospho-p38 MAPK (Thr180/Tyr182) and phospho-p42/44 MAPK (Thr202/Tyr204) in Huh7.5 cell lysates prepared after 48 h of transfection of SARS-CoV-2 spike gene construct with or without Candesartan cilexetil treatment. Expression level of actin in each lane is shown as a total protein loading control for comparison. **(B)** Western blot analysis of phospho-p38 MAPK (Thr180/Tyr182) and phospho-p42/44 MAPK (Thr202/Tyr204) in A549 cell lysates prepared after 48 h of transfection of SARS-CoV-2 spike gene construct with or without Candesartan cilexetil treatment. Expression level of actin or tubulin in each lane from the same gel is shown as a total protein load for comparison.

To verify the involvement of AT1 signaling in MAPK activation, we used the AT1 receptor antagonist Candesartan cilexetil in Huh7.5 and A549 cell lines expressing SARS-CoV-2 spike protein. Candesartan cilexetil binds to the angiotensin II receptor type 1 to block the binding of angiotensin II to its target receptor expressed in vascular and other cell types to regulate blood pressure and fluid retention [26]. Our results indicated that the presence of viral spike protein induced phospho-p38 MAPK (Thr180/Tyr182) and phospho-p42/44 MAPK (Thr202/Tyr204), and inhibited IκBα expression, while introduction of Candesartan cilexetil modulated the effect spike has upon the expression of these proteins (Fig 3A and 3B). These results provided evidence that SARS-CoV-2 spike protein expression in epithelial cells activates MAPK signaling cascade by induction of the AT1 axis.

### Presence of SARS-CoV-2 spike protein stimulates IL-6 and soluble IL-6R production

**Fig 4:**
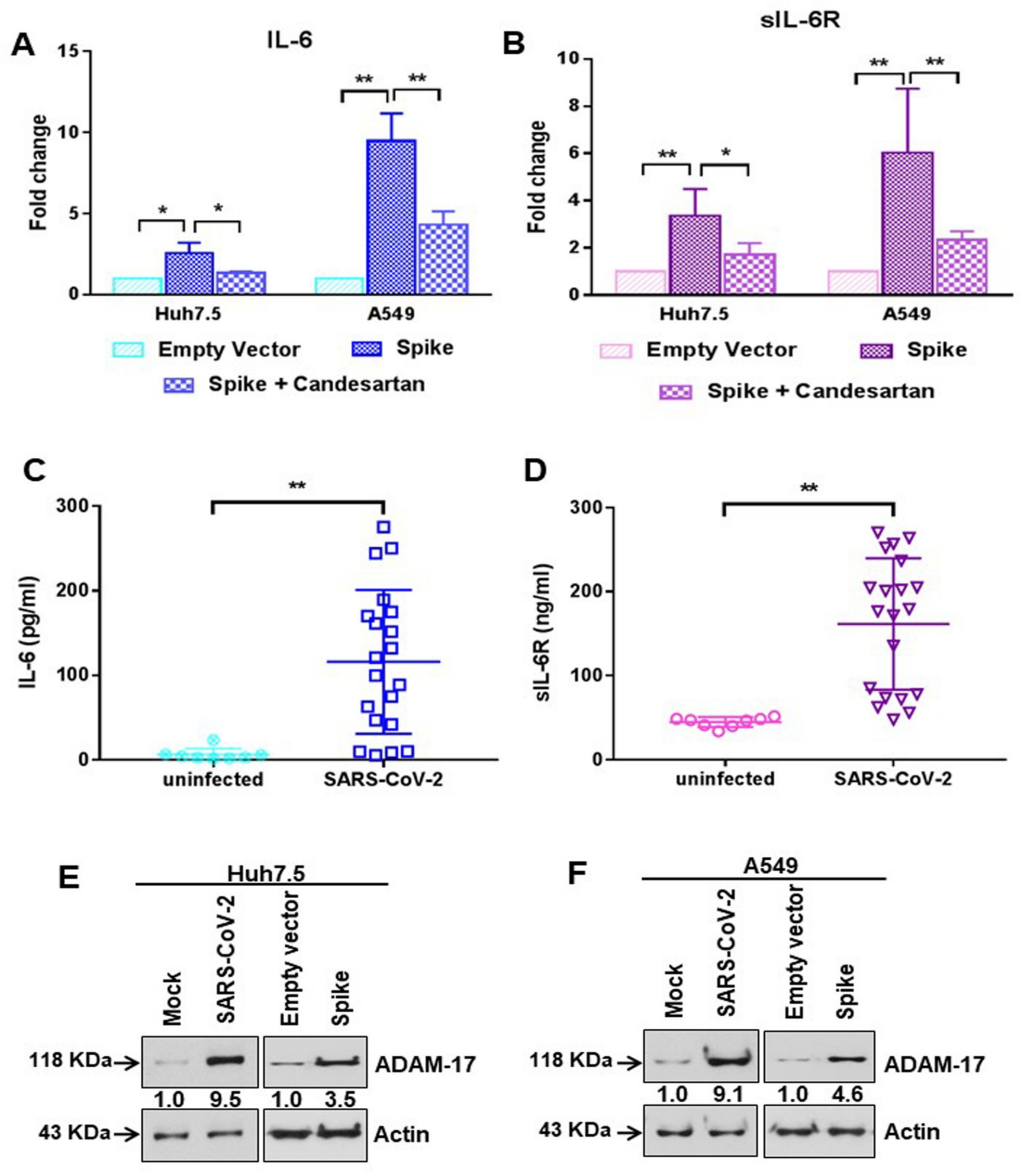
SARS-CoV-2 spike protein stimulates IL-6 and soluble IL-6R production. **(A)** The extra-cellular level of IL-6 was measured by ELISA from culture supernatant of Huh7.5 and A549 cells after transfection of SARS-CoV-2 spike gene construct with or without Candesartan cilexetil treatment. **(B)** The extra-cellular level of soluble IL-6R was similarly measured by ELISA from culture supernatant of Huh7.5 and A549 cells after transfection of SARS-CoV-2 spike gene construct with or without Candesartan cilexetil treatment as shown in panel A. The results are presented as means ± standard deviations. ‘*’ and ‘**’ represent statistical significance *p*<0.05 and *p*<0.005, respectively. **(C)** The level of IL-6 was measured by ELISA from the serum samples of SARS-CoV-2 infected patients (n = 20) and uninfected healthy volunteers (n = 8). **(D)** The level of soluble IL-6R was similarly measured by ELISA from the serum samples of SARS-CoV-2 infected patients (n = 20) and uninfected healthy volunteers (n = 8) as shown in panel C. The results are presented as means ± standard deviations. ‘*’ and ‘**’ represent statistical significance *p*<0.05 and *p*<0.005, respectively. **(E, F)** Western blot analysis of ADAM-17 expression in Huh7.5 and A549 cell lysates prepared after 48 h of mock-treated or infected with SARS-CoV-2 virus, or transiently transfected with empty vector or SARS-CoV-2 spike gene construct. Expression level of actin in each lane is shown as a total protein loading control for comparison.

**Fig 5:**
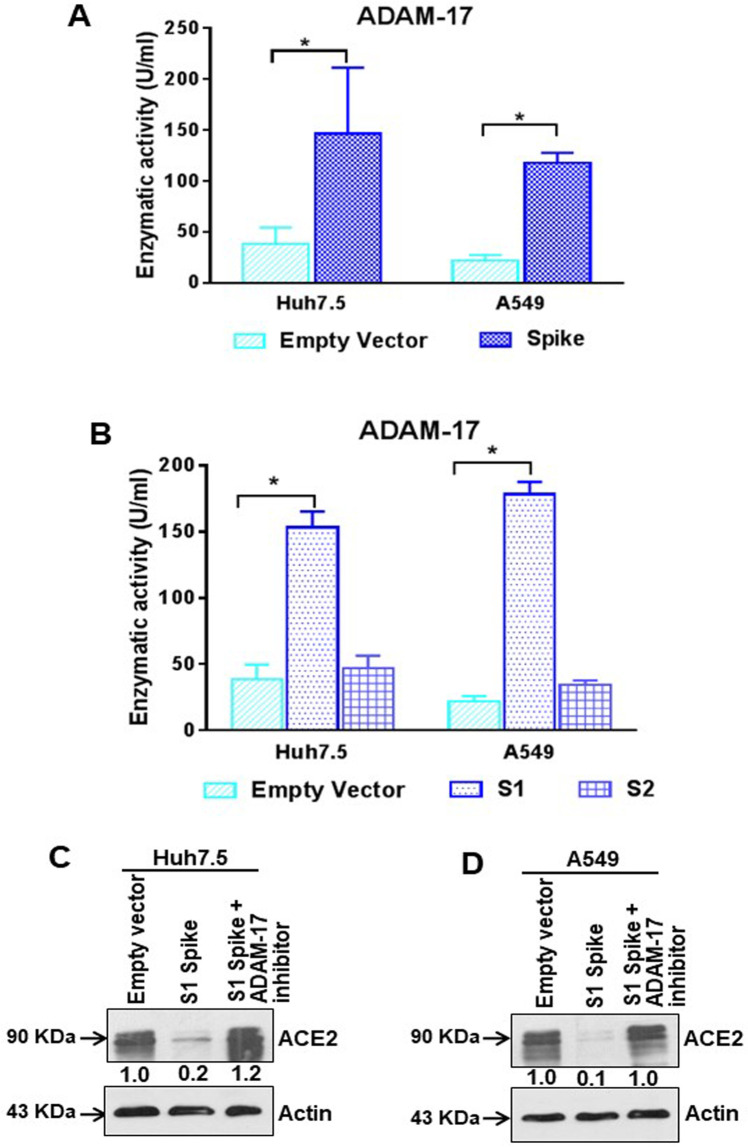
SARS-CoV-2 spike protein regulates ADAM-17 activity. **(A)** ADAM-17 enzymatic activity was measured from crude extracts of Huh7.5 and A549 cells after transfection of SARS-CoV-2 spike gene constructs. **(B)** ADAM-17 enzymatic activity was measured from crude extracts of Huh7.5 and A549 cells after transfection of SARS-CoV-2 spike S1 or S2 gene construct or empty vector. The results are presented as mean ± standard deviation. ‘*’ represent statistical significance (*p*<0.05). **(C, D)** Western blot analysis of ACE2 expression in Huh7.5 and A549 cell lysates prepared after 48 h of transfection of empty vector or SARS-CoV-2 spike S1 gene construct in the presence or absence of ADAM-17 inhibitor. Expression level of actin in each lane from same gel is shown as a total protein loading control for comparison.

The pro-inflammatory cytokine IL-6 is involved in the development of chronic inflammatory diseases. IL-6 synthesis is regulated through the activation of the MAPK/NF-κB pathway [27]. IL-6 is one of the major inflammatory molecules involved in SARS-CoV-2 infection. We performed an ELISA to detect the level of IL-6 in the culture supernatant in Huh7.5 and A549 cells transfected with SARS-CoV-2 spike gene, and observed an increase in extracellular IL-6 levels in spike transfected cells (Fig 4A). Further, treatment with Candesartan cilexetil to inhibit the AT1 signaling pathway led to reduced secreted IL-6 levels (Fig 4A). Interestingly, we noticed that extra-cellular soluble IL-6R levels were also increased in the culture supernatants of spike transfected Huh7.5 and A549 cells, while introduction of Candesartan cilexetil reduced the level of soluble IL-6R (Fig 4B). To verify the relevance of these *in vitro* observations in COVID-19 patient samples, we measured twenty SARS-CoV-2 virus infected patient sera for detection of IL-6 and soluble IL-6R levels. We found that both IL-6 and soluble IL-6R concentrations were increased in virus infected samples, as compared to eight uninfected healthy control sera (Fig 4C and 4D). The induction of ADAM-17 metalloprotease is responsible for the generation of soluble IL-6R by cleavage of transmembrane IL-6R. ADAM-17-mediated shedding of cytokine receptors causes desensitizing of the cell to their ligands. Additionally, the liberated soluble receptor IL-6R acts as an activator via trans-signaling [28]. Here, we observed an induction of ADAM-17 expression (antibody recognizing the precursor form only) in SARS-CoV-2 infected or viral spike protein transfected Huh7.5 and A549 cells (Fig 4E and 4F). Enzymatic activity of ADAM-17 was measured from epithelial cells after SARS-CoV-2 spike gene transfection. ADAM-17 activity was significantly elevated in viral spike protein expressing Huh7.5 or A549 cells, as compared to empty vector transfected control cells (Fig 5A). We also observed ADAM-17 activity was higher in S1 region of spike protein transfected cells, but not with the S2 region (Fig 5B). Treatment with ADAM-17 inhibitor protected ACE2 cleavage in the presence of SARS-CoV-2 spike S1 region (Fig 5C and 5D). This result indicated that enhanced ADAM-17 activity is responsible for ACE2 cleavage, similar to the earlier reports on SARS-CoV spike protein expressing cells [29, 30]. Thus, our study demonstrated that the presence of SARS-CoV-2 spike protein leads to increased IL-6 secretion, and induces ADAM-17 mediated release of soluble IL-6R from epithelial cells.

### Inhibition of STAT3 Tyr705 phosphorylation in SARS-CoV-2 spike protein expressing cells

**Fig 6:**
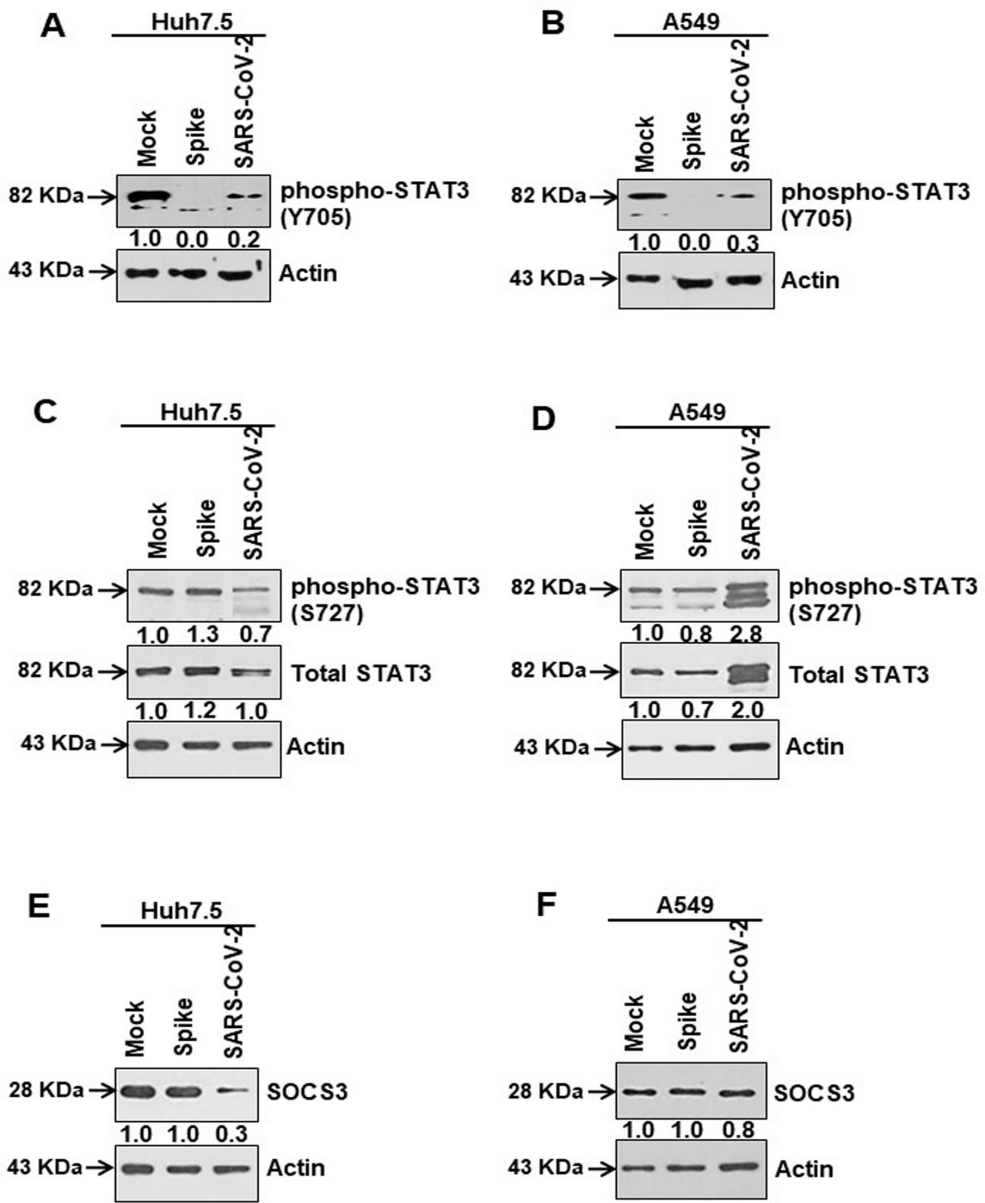
SARS-CoV-2 spike protein causes inhibition of tyrosine phosphorylation of STAT3. **(A, B)** Western blot analysis of phospho-STAT3 (Tyr705) and total STAT3 expression in Huh7.5 and A549 cell lysates prepared after 48 h of mock-treated or transient transfection of SARS-CoV-2 spike gene constructs or infection with SARS-CoV-2 virus. **(C, D)** Western blot analysis of phospho-STAT3 (Ser727) and total STAT3 expression in Huh7.5 and A549 cell lysates prepared after 48 h of mock-treated or transiently transfection of SARS-CoV-2 spike gene construct or infection with SARS-CoV-2 virus. **(E, F)** Western blot analysis of SOCS3 expression in Huh7.5 and A549 cell lysates prepared after 48 h of mock-treated or transiently transfected with SARS-CoV-2 spike constructs or infected with SARS-CoV-2 virus. Expression level of actin in each lane from the same gel is shown as a total protein loading control for comparison.

Released extracellular IL-6 generally binds to its receptor, where homodimer complexes of gp130 are formed leading to downstream Janus kinases/signal transducers and activators of transcription 3 (Jak/STAT3) are activated. Functional activation of STAT3 occurs via phosphorylation of the Tyr705 residue, resulting in translocation of STAT3 to the nucleus [31]. We observed an inhibition of STAT3 Tyr705 phosphorylation in SARS-CoV-2 infected or viral spike protein transfected Huh7.5 and A549 cells when compared to total STAT3 expression (Fig 6A and 6B). Phosphorylation at Ser727 is another important site of STAT3 which determines mitochondrial translocation, was unaffected when compared to total STAT3 expression (Fig 6C and 6D). Minor differences in band intensities observed could be due to total protein load in lanes. Up-regulation of suppressor of cytokine signaling 3 (SOCS3) is an indicator of STAT3 transcriptional activation [32]. Our western blot data showed SOCS3 expression decreased or was not enhanced in the epithelial cells expressing SARS-CoV-2 spike protein (Fig 6E and 6F). Thus, our data suggested that SARS-CoV-2 spike protein induces IL-6 secretion, which remains unresponsive to IL-6 classical signaling for ADAM-17 cleavage.

### Activation of IL-6 trans-signaling from SARS-CoV-2 spike protein expressing epithelial cells forms an inflammatory circuit in endothelial cells

**Fig 7:**
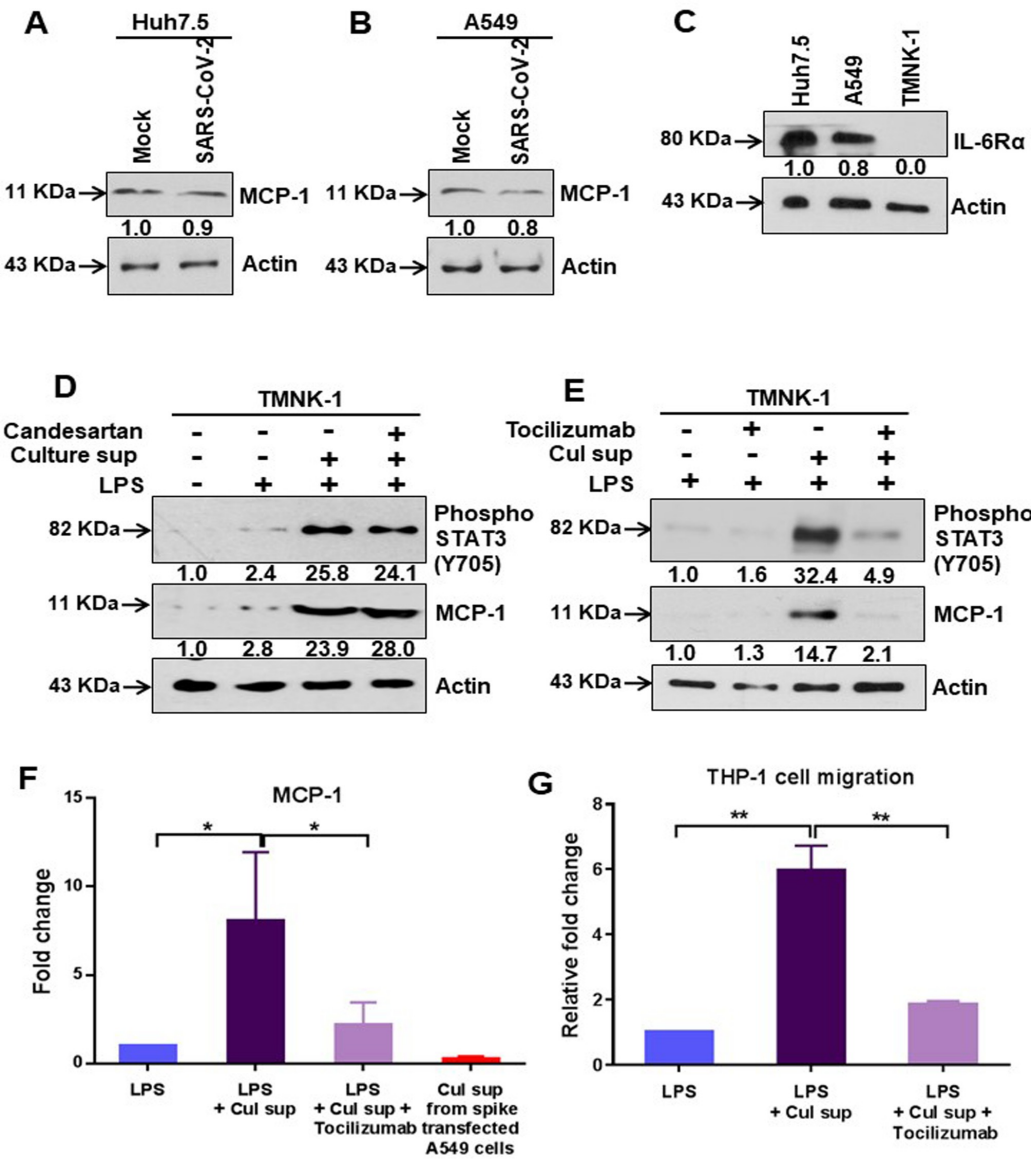
IL-6 trans-signaling induces MCP-1 expression. **(A, B)** Western blot analysis of MCP-1 expression in Huh7.5 and A549 cell lysates prepared after 48 h of mock-treated or infected with SARS-CoV-2 virus. **(C)** Western blot analysis for IL-6Rα expression in Huh7.5, A549, or TMNK-1 cell lysates. **(D)** Western blot analysis of phospho-STAT3 (Tyr705) and MCP-1 expression in TMNK-1 liver endothelial cell lysates prepared after treated with or without culture supernatant from SARS-CoV-2 spike gene expressed A549 cells in presence or absence of Candesartan cilexetil and LPS. **(E)** Western blot analysis for phospho-STAT3 (Tyr705) and MCP-1 expression status in TMNK-1 liver endothelial cell lysates prepared after treatment with LPS and culture supernatant from SARS-CoV-2 spike gene expressed A549 cells in the presence or absence of Tocilizumab. Expression level of actin in each lane from the same gel is shown as a total protein load for comparison. **(F)** The extra-cellular level of MCP-1 was measured by ELISA in culture supernatant of TMNK-1 cells after treatment with LPS alone or together with culture supernatant from SARS-CoV-2 spike gene expressing A549 cells in the presence or absence of Tocilizumab. The MCP-1 expression level in the culture supernatant from SARS-CoV-2 spike gene expressing A549 cells was also detected. **(G)** Comparative analysis of cellular migration of human monocytes (THP-1) in the presence of culture supernatant from TMNK-1 cells treated with LPS, and from culture supernatant of SARS-CoV-2 spike gene expressed A549 cells in the presence or absence of Tocilizumab. The results are presented as mean ± standard deviation. ‘*’ (*p*<0.05) and ‘**’ (*p*<0.005) represent statistical significance.

We have observed that epithelial cells expressing SARS-CoV-2 spike protein were unable to induce STAT3 mediated transcriptional activity, which is necessary to induce monocyte chemoattractant protein-1 (MCP-1) expression [33]. To revisit our observation, we examined MCP-1 in virus infected Huh7.5 and A549 cells by western blot analysis. As expected, there was no significant change in MCP-1 expression in both epithelial cell lines indicating an inhibitory effect on STAT3 Tyr705 phosphorylation by virus infection (Fig 7A and 7B). Furthermore, we examined whether IL-6/soluble IL-6R released in cell culture supernatant from spike gene transfected epithelial cells can promote IL-6 trans-signaling activity upon endothelial cells as a model expressing a weak IL-6R. Endothelial cells express gp130, but not transmembrane IL-6R, and produce IL-6 and MCP-1 upon exposure to several stimuli [34]. TMNK-1 liver endothelial cells poorly expressed IL-6Rα (Fig 7C). TMNK-1cells stimulated by LPS were treated with or without culture supernatant from SARS-CoV-2 spike expressing A549 cells in the presence or absence of an AT1 receptor antagonist. Our results showed increased phospho-STAT3 (Tyr705) expression and MCP-1 expression in TMNK-1 cells, which was not inhibited by AT1 receptor blockage (Fig 7D). To further verify the involvement of IL-6 in a trans-signaling mechanism, we incubated LPS stimulated TMNK-1 cells with IL-6 signaling blocker Tocilizumab and culture supernatant from SARS-CoV-2 spike expressing A549 cells. Western blot analysis suggested an increased phospho-STAT3 (Tyr705) and MCP-1 expression in TMNK-1 cells treated with culture supernatant from SARS-CoV-2 spike expressing A549 cells, and this was significantly suppressed by Tocilizumab (Fig 7E). We also analyzed the level of MCP-1 by ELISA in culture supernatant of TMNK-1 cells following treatment with LPS and spike transfected A549 culture medium. The MCP-1 level was elevated when treated with culture supernatant from SARS-CoV-2 spike expressing cells; and significantly reduced upon addition of Tocilizumab (Fig 7F). The biological activity of secreted MCP-1 was examined by a chemotaxis assay on human monocytes (THP-1). A significant level of THP-1 cell migration was observed in the presence of culture supernatant from TMNK-1 where MCP-1 expression was elevated (Fig 7G). These results provided further evidence that SARS-CoV-2 spike protein activates IL-6 trans-signaling, but not classical signaling in epithelial cells leading to a hyper-inflammatory response.

**Fig 8:**
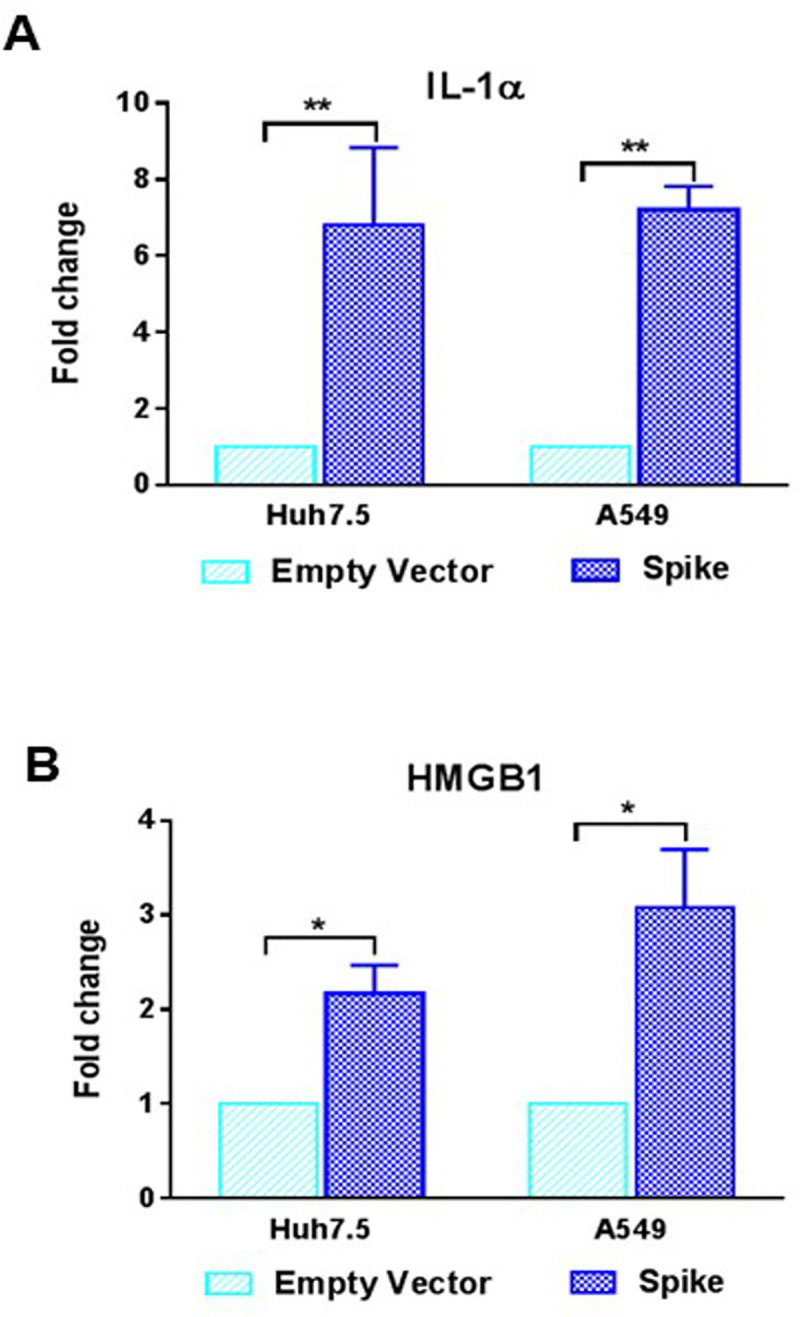
SARS-CoV-2 spike protein induces alarmin secretion from epithelial cells. **(A, B)** The extra-cellular level of IL-1α and HMGB1 were measured by ELISA from culture supernatant of Huh7.5 and A549 cells after transfection of SARS-CoV-2 spike gene construct or empty vector as a control for comparison. The results are presented as mean ± standard deviation. ‘*’ (*p*<0.05) and ‘**’ (*p*<0.005) represent statistical significance.

Alarmins are released from deleterious response in the host provoking an uncontrolled inflammatory activity. This group of proteins includes IL-1α and the high-mobility group box 1 protein (HMGB1) [35]. We measured the release of alarmins in SARS-CoV-2 spike transfected Huh7.5 and A549 cells from the culture supernatants. A 6–8 fold increase in the secretion of IL-1α and a 2–4 fold increase in the secretion of HMGB1 were noted (Fig 8A and 8B). These results indicated that the presence of SARS-CoV-2 spike protein in the epithelial cells causes potential deleterious effect which may have shared through the cellular environments.

## Discussion

We investigated the contribution of SARS-CoV-2 spike protein on the renin–angiotensin system. SARS-CoV-2 infection or ectopic spike protein expression in human epithelial cells inhibited ACE2 expression, leading to increased angiotensin II and AT1 receptor expression. An earlier study suggested that angiotensin II upregulates AT1 receptor expression [36]. Our *in vitro* observations are consistent with the recent report that the angiotensin II levels in plasma from SARS-CoV-2 virus infected patients are markedly elevated and associated with viral load and lung injury [37]. SARS-CoV infection associated severe acute lung pathologies also correlates with a reduced ACE2 expression [38, 39]. To our knowledge, the present study uniquely demonstrates that the presence of SARS-CoV-2 spike protein, or specifically its S1 region, induces AT1 signaling in human epithelial cells.

Our study demonstrated that SARS-CoV-2 spike protein was associated with the up-regulation of AT1 signaling which led to the induction of transcriptional regulatory molecules, such as NF-κB, c-Fos, and MAPK activation. Cytokine storm during SARS-CoV-2 infection is a possible mechanism of morbidity and mortality, and IL-6 is one of the major inflammatory molecules involved in COVID-19 [9,10]. Spike protein induced the generation of IL-6 in cultured cells as well as in COVID-19 positive patient sera, and AT1 receptor antagonist resulted in down-regulation of MAPK activation as well as IL-6 release. Hadjadj et al. [8] reported that increased IL-6 is one of the major players in the exacerbated inflammatory response associated with COVID-19 disease, and up-regulation of the NF-κB pathway may have a role in disease pathogenesis. SARS-CoV-2 infection causes an imbalance of the renin-angiotensin system, leading to an increased concentration of pro-inflammatory molecules. We now have a more complete picture of the sequential mechanistic steps of SARS-CoV-2 infection in epithelial cells. A retrospective cohort study with COVID-19 patients also suggests that angiotensin II receptor blocker medications are associated with a reduced risk of hospitalization and admission to the ICU [40].

**Fig 9:**
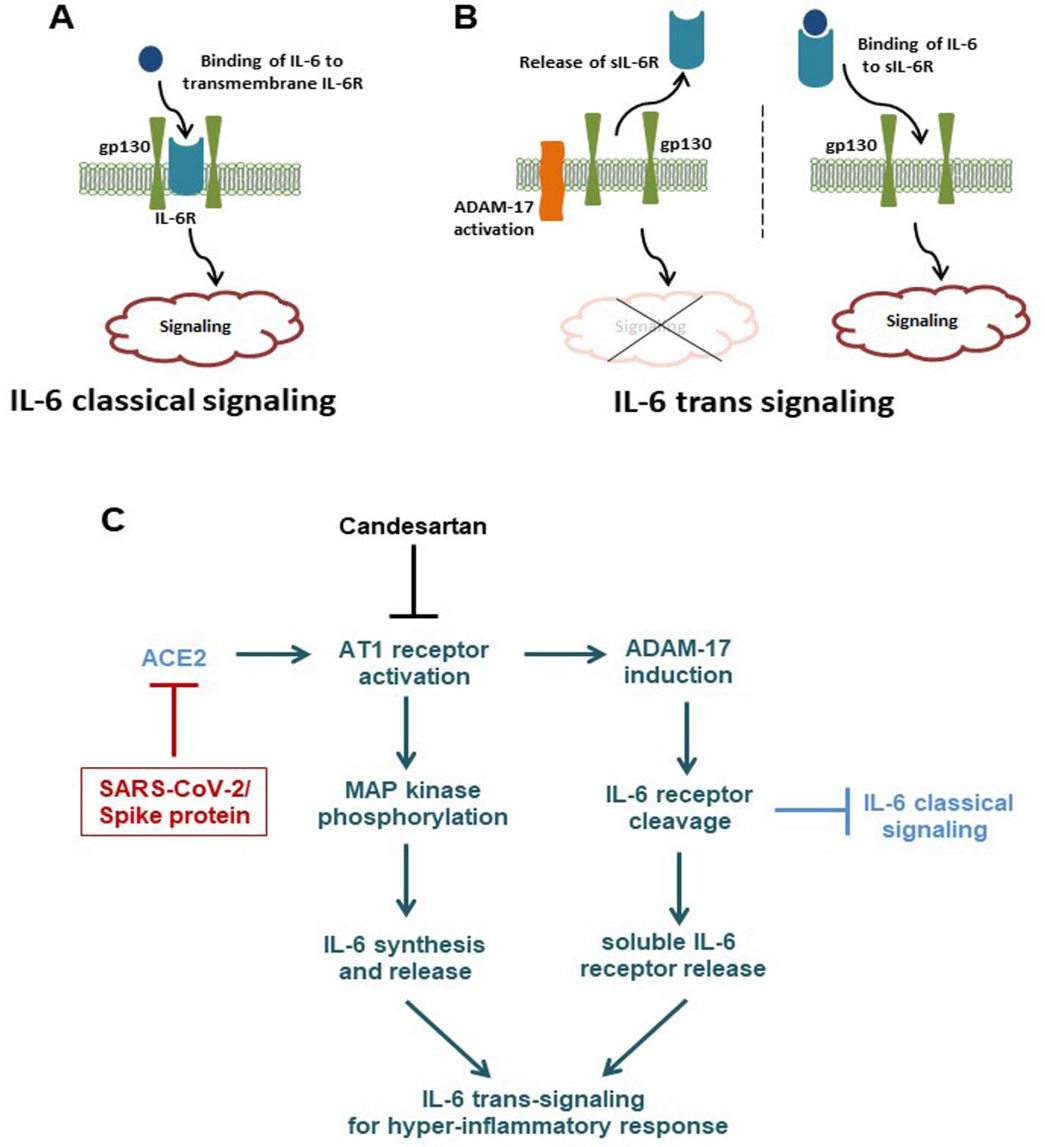
**(A, B)** Overview of IL-6 mediated classical and trans-signaling mechanisms. **(C)** Schematic presentation of molecular changes occurring upon SARS-CoV-2 infection or spike protein expression in epithelial cells.

IL-6 signaling is triggered when bound to the transmembrane IL-6 receptor. Alternatively, IL-6 may bind to a soluble form of the IL-6 receptor and activate STAT3 signaling in cells that express gp130, exclusive of transmembrane IL-6R. In general, classic IL-6 signaling is responsible for the anti-inflammatory properties of IL-6, whereas trans-signaling is responsible for the pro-inflammatory actions of IL-6 [41]. While soluble IL-6R can be released following alternative splicing of the IL-6R mRNA, most circulating soluble IL-6R is generated by the ADAM-17 mediated proteolysis of the transmembrane IL-6R [21, 28] (Fig 9A). Enhanced IL-6 trans-signaling is observed in chronic obstructive pulmonary disease (COPD), rheumatoid arthritis, inflammatory bowel disease, and other autoimmune diseases [42]. Higher levels of soluble IL-6R have been found in the sputum and broncho-alveolar lavage (BAL) fluid of people with asthma or COPD when compared to healthy controls [43, 44]. However, a significant elevation of soluble IL-6R has been noticed in the serum of HIV infected patients, as well as, in influenza A virus infection [45, 46]. We observed an increased level of soluble IL-6R in SARS-CoV-2 infected patients. A recent study also suggested elevated levels of IL-6 and its soluble receptor in COVID-19 patients [47]. Interestingly, a major difference in acute (6 sera collected at 1–2 weeks) and post-infection (14 sera collected at 4–6 weeks) of SARS-CoV-2 infected patients for IL-6/soluble IL-6R levels was not observed in our results, and analyzing large sample numbers will provide a more definite conclusion. Our study also revealed that the presence of SARS-CoV-2 spike protein in epithelial cells induces extra-cellular soluble IL-6R by activation of ADAM-17 protease. On the other hand, SARS-CoV infection is known to de-phosphorylate STAT3 tyrosine 705 in epithelial cells [48]. This information coincided with our observations in SARS-CoV-2 infected or ectopic spike protein expressing human epithelial cells where tyrosine phosphorylation was inhibited. Thus, inhibition of STAT3 tyrosine phosphorylation was unable to induce monocyte chemoattractant protein MCP-1 expression in epithelial cells, due to the importance of STAT3 as a transcriptional inducer for MCP-1. However, introduction of culture supernatant from SARS-CoV-2 spike expressing epithelial cells resulted in the induction of STAT3 tyrosine phosphorylation as well as MCP-1 expression in endothelial cells, where transmembrane IL-6R is poorly expressed (Fig 9B). Therefore, activation of IL-6 trans-signaling from SARS-CoV-2 spike protein expression in epithelial cells governs an inflammatory circuit in endothelial cells. Recent studies showed that MCP-1 was also significantly elevated in COVID-19 patients in both mild and severe cases, when compared to healthy controls in causing an aggravated inflammatory response [7, 49]. Release of IL-6 and soluble IL-6R in circulation or activation of IL-6 trans-signaling in SARS-CoV-2 infected patients is speculated to act as a chemoattractant for monocyte/macrophage with the potential for tissue damage via bystander mechanisms [50], and our present results support this hypothesis. A recent study suggested that IL-6 is one of the cytokines responsible for immune dysregulation or macrophage activation syndrome in severe COVID-19 patients, and the use of Tocilizumab partially rescued SARS-CoV-2 associated immune dysregulation [51]. We speculate that SARS-CoV-2 infection in pulmonary epithelial cells induces IL-6 trans-signaling for secretion of chemokines; like MCP-1, from pulmonary vascular endothelial cells, and attract monocytes/macrophages to create a hyper-inflammatory state leading to pulmonary edema and a disturbance of oxygen exchange or acute respiratory distress syndrome.

## Materials and methods

### Ethics Statement

Coded clinical serum samples from twenty adult patients positive for SARS-CoV-2 infection and eight serum samples from uninfected healthy volunteers were obtained from Saint Louis University Hospital COVID-19 repository (Institutional Review Board Approval Numbers 26646 and 27790). Written or verbal consents were obtained from the patients and volunteers.

### Cell culture and virus infection

Transformed human lung epithelial cells (A549), liver epithelial cells (Huh7.5), and liver sinusoidal endothelial cells (TMNK-1) (kindly provided by A. Soto-Gutierrez, University of Pittsburg, PA) were cultured in Dulbecco’s modified Eagle’s medium (DMEM) (Hyclone) containing 10% fetal bovine serum (FBS) (Sigma), 100 U of penicillin/ml, and 100 mg of streptomycin/ml (Sigma). The cells were maintained in a humidified atmosphere at 37°C with 5% CO_2_. For virus infection, the cells were cultured in DMEM containing 2% FBS in 25 cm^2^ T-flask or 6-well plate at ~60% confluency. SARS-CoV-2 isolate (USA-WA1/2020, kindly provided by BEI Resources) from Wuhan, China, was grown in Vero E6 cells. Early passaged virus was used for infection of other cell lines at a multiplicity of infection of 0.5 (moi = 0.5). All live virus related experiments were performed in our Institutional Biosafety Committee (IBC) approved P3 laboratory facility. Infected cells were lysed after 48 h of infection and lysates were used for further experiments.

### Transient transfection

Cells were sub-cultured in 6-well plate at ~60% confluency and transfected with plasmid DNA (pcDNA3.1-SARS-CoV-2-Spike MC-0101087-5834, kindly provided from BEI Resources), pCMV3-SARS-CoV-2-Spike S1 region or pCMV3-SARS-CoV-2-Spike S2 region (Sino Biological), and empty vector construct (500 ng/plate) using Lipofectamine 3000 (Life Technologies) following the manufacturer’s instruction. Cell lysates were prepared after 48 h of transfection for analyses. AP1-luciferase construct was obtained from commercial source (Addgene) and used in luciferase assay.

### Inhibitor treatment

AT1 receptor antagonist Candesartan cilexetil (Tocris) was used in this study. The cells were treated with the Candesartan cilexetil (dissolved in DMSO) at 1 μM concentration for 20 h following earlier reports [52, 53]. ADAM-17 inhibitor GM6001 (Enzo Life Sciences) was dissolved in DMSO and cells treated at 100 nM concentration for 20 h [54].

### Western blot analysis

Cell lysates were electrophoresed to resolve proteins by SDS-PAGE, transferred onto a nitro-cellulose membrane, and blocked with 4% non-fat dry milk. The membrane was incubated at 4°C overnight with specific primary antibody, followed by a secondary antibody conjugated with horseradish peroxidase. The protein bands were detected by chemiluminescence (Life Technologies). The blot from the same run was reprobed with β-actin (Sigma) or α-tubulin (Santa Cruz Biotechnology) HRP conjugated antibody to compare protein load in each lane. Commercially available antibodies for ACE2, AT1 receptor, c-Fos, IκBα, ADAM-17, MCP-1 and IL-6Rα were procured from Santa Cruz Biotechnology; phospho-p38 MAPK (Thr180/Tyr182), phospho-p42/44 MAPK (Thr202/Tyr204), phospho-NF-κB (Ser276), SOCS3, phospho-STAT3 (Tyr705), phospho-STAT3 (Ser727), total STAT3 were procured from Cell Signaling Technologies; and SARS-CoV-2 spike protein (Sino Biological) were also procured for western blot analyses.

### ELISA

Patient serum samples and cell culture supernatants from SARS-CoV-2 spike protein transfected cells were analyzed for the presence of secreted IL-6 (Sigma), soluble IL-6R (Life Technologies) IL-1α (Sigma) and HMGB-1 (Novus Biologicals) using ELISA kits following the manufacturer’s instructions. We also analyzed MCP-1 level by ELISA in culture supernatants of LPS stimulated TMNK-1 cells and from SARS-CoV-2 spike expressing A549 cell culture medium in the presence or absence of Tocilizumab (Absolute Antibody).

### ADAM-17 enzymatic assay

ADAM-17 enzymatic activity was analyzed by fluorometric cleavage of specific substrate [55]. Briefly, crude enzyme extract was prepared from cell suspension followed by pulse sonication and centrifugation. A total 100 μl of reaction mixture was prepared in assay buffer containing 20 μl of crude cell extract and 10 μM fluorogenic substrate Mca-PLAQAV-Dpa-RSSSR-NH_2_ (Enzo Life Sciences). A change in fluorescence was measured after addition of substrate at 5 minutes time interval up to 40 minutes in a Synergy Mx Microplate Reader (BioTek Instruments, Inc.). The rate of enzyme activity was calculated using recombinant human ADAM-17 as a positive control. Total protein estimation was performed using BCA kit (Thermo Fisher) following supplier’s instruction.

### Chemotaxis assay for MCP-1

Chemotactic activity of MCP-1 was performed by cellular migration assay of THP-1 cells using QCM (5 μm) fluorometric 24-well chemotaxis cell migration assay kit (Sigma).

### Statistical analysis

Graph Pad Prism 7 was used to analyze the experimental data. All experiments were performed at least three times for reproducibility. The results are presented as mean ± standard deviation. Non-parametric Mann-Whitney U test and paired two-tailed t-test analyses were performed to compare the mean values between the two groups. Statistical significance was considered as *p* <0.05.
